# Novel Findings of Retinal and Choroidal Features Utilizing Optical Coherence Tomography Angiography Analysis in Patients With Autoimmune Posterior Uveitis

**DOI:** 10.3389/fmed.2021.801036

**Published:** 2022-01-11

**Authors:** Junhui Shen, Jinfeng Kong, Si Chen, Xin Liu, Yan Teng, Hailan Wu, Lijuan Wang, Manman Wu, Zhaoan Su, Lei Feng

**Affiliations:** Eye Center, Second Affiliated Hospital, School of Medicine, Zhejiang University, Hangzhou, China

**Keywords:** optical coherence tomography angiography, quantitative measurements, uveitis, vessel density, inflammation

## Abstract

**Purpose:** To analyze the quantitative parameters acquired by optical coherence tomography angiography (OCTA) in patients with autoimmune posterior uveitis.

**Methods:** OCTA images of 65 eyes affected with uveitis and 65 normal control (NC) eyes were obtained. The central macular thickness (CMT), retinal thicknesses, foveal avascular zone (FAZ) area, foveal density 300 μm (FD300), and vascular density (VD) were compared among acute uveitic eyes, chronic uveitic eyes, and NC eyes. VDs were evaluated in the choriocapillaris, outer retina, optic disk, whole and parafovea superficial capillary plexus (SCP), and whole and parafovea deep capillary plexus (DCP). Correlation analysis was used to analyze the relationship between LogMAR best-corrected visual acuity (BCVA) and quantitative parameters from OCTA.

**Results:** Compared with NC eyes, the CMT and retinal thicknesses were increased significantly in eyes with uveitis (*p* < 0.05, respectively). No significant difference was observed in the FAZ area. FD300, VDs in the optic disk, SCP, and DCP both in whole image and parafovea, choriocapillaris were significantly decreased in uveitis eyes (*p* < 0.05, respectively) compared with NC eyes, only the acute group had decreased VD of the outer retina and choriocapillaris compared with the NC group (*p* < 0.05). Moreover, quantitative parameters of OCTA showed a significant correlation with LogMAR BCVA in the patients with uveitis. Whole VD DCP was the best predictive factor for BCVA in the patients with uveitis.

**Conclusion:** Quantitative measurement by OCTA is a promising strategy for objective assessment of autoimmune posterior uveitis.

## Introduction

Optical coherence tomography angiography is a new non-invasive fundus imaging technique that can be used to acquire information about retinal and choroidal blood flow with high resolution *in vivo* (1). The high resolution enables the display of the signals of the retinal capillary network and choroidal capillary network in different layers. OCTA has unique advantages in the detection of retinal or choroidal vascular changes, the measurement of foveal avascular zone (FAZ), the quantification of vascular density (VD) in the inner retina, outer retinal circulation, or choriocapillaris. The technique has already been applied in the study of glaucoma (2) and various kinds of retinopathy, such as central serous choroidopathy (3), choroidal neovascularization (4), polypoid choroidal vasculopathy (PCV) (5), and diabetic retinopathy (6).

When the uvea is in an inflammatory state, the blood flow of the short posterior ciliary artery and the choroid are decreased, and the morphology and the function of retinal blood vessels are also compromised. To date, alterations of the retinal vessels in anterior, posterior, and panuveitis have been studied. OCTA has also proved to be an effective diagnostic tool in birdshot chorioretinopathy (7), multifocal choroiditis (8, 9), punctate inner choroidopathy (PIC) (10), acute macular neuroretinopathy (11), multiple evanescent white dot syndrome (12), acute posterior multifocal placoid pigment epitheliopathy (APMPPE) (13), and serpiginous-like choroiditis (14). Several researchers have revealed that OCTA can provide quantitative analysis for uveitis. Koca et al. found that retinal VD decreased and the perifoveal microvascular network changed in ocular-involved Behçet's (15). Liang et al. found that deep capillary plexus (DCP) VD was significantly lower in patients with Vogt–Koyanagi–Harada disease (VKH) than in normal controls (NCs), and that DCP VD was associated with a visual outcome (1). So far, published studies on the quantitative analysis of OCTA in uveitis are very limited, especially in patients with posterior segment-involved autoimmune uveitis. There is a lack of investigation about the vascular beds below the retinal pigmented epithelium (RPE) complex, quantitative analysis of the outer retina, or choriocapillaris abnormalities.

In this study, retinal and choroidal microvasculature changes in patients with uveitis, and healthy controls were studied. We aimed to summarize OCTA features from uveitis at different stages to explore the potential clinical values of quantitative OCTA results and to assess the values of OCTA measurement in the diagnosis, follow-up, and prognosis of patients with autoimmune posterior uveitis.

## Materials and Methods

### Study Participants

The present study employed a retrospective approach. Patients diagnosed with autoimmune posterior uveitis and NCs who were at the Second Affiliated Hospital of Zhejiang University School of Medicine between May 2020 and September 2020 were enrolled. The consent procedure and study protocol followed the tenets of the Declaration of Helsinki and were approved by the Institutional Review Board of the Second Affiliated Hospital of Zhejiang University School of Medicine. Written informed consent was obtained from the patients.

### Inclusion Criteria

All autoimmune posterior uveitis cases were diagnosed based on the American Uveitis Society's revised international criteria through clinical examination techniques such as slit-lamp evaluations of the anterior segment, color fundus, OCTA, fluorescein angiography, and indocyanine green angiography if needed. Laboratory examinations, including white blood cell counts, serologic examinations for syphilis, and tuberculin tests, were assessed to determine the underlying cause of uveitis. Patients with posterior uveitis were grouped into two stages according to the classic Moorthy criteria: (1) acute uveitic stage: disease course within 3 months or (2) chronic stage: disease course exceeding 3 months. In cases of bilateral uveitis, only one eye was chosen randomly in the analysis.

During the same period, age-matched healthy volunteers without a history of ocular inflammation, injury, surgery, or other remarkable ocular diseases were recruited. Only one eye was chosen randomly in the analysis.

Medical records of the patients and NCs were reviewed retrospectively, including sex, age, uveitis diagnosis, OCTA images, and best-corrected visual acuity (BCVA).

### Exclusion Criteria

We excluded patients with infectious posterior uveitis. Eyes with retinal or choroidal vascular disease not associated with uveitis (e.g., diabetic retinopathy, retinal vascular occlusion, and age-related macular degeneration), masquerade syndrome, glaucoma, and high myopia were excluded. The subjects who were unable to fixate or who had significant media opacities were excluded. OCTA images with poor quality, such as projection artifacts from vessels located above the plane of the image or an overly dark image filled with extremely thick outer choroidal vessels, were excluded.

### OCTA Image Analysis

All patients and NCs were imaged with the AngioVue Imaging System (RTVue XR Avanti; Optovue, Inc., Fremont, CA) (16) by the same experienced examiner. OCTA image analysis covering the 4.5 mm × 4.5 mm area centered on the papilla and the 6 mm × 6 mm area centered on the fovea was performed. The qualities of the images were graded automatically by the AngioAnalytics software (Optovue, Inc.) from Q1 (lowest quality) to Q10 (highest quality), and only images with qualities of Q7 or higher were included in the analysis. A CUSTOM function in the AngioVue software (version 2018.1.0.43; Optovue, Inc.) was used to measure retinal nerve fiber layer (RNFL), CMT, retinal thickness, FAZ area, FD300, and VDs in the optic disk, superficial capillary plexus (SCP), DCP, outer retina, and choriocapillaris.

**Figure 1:**
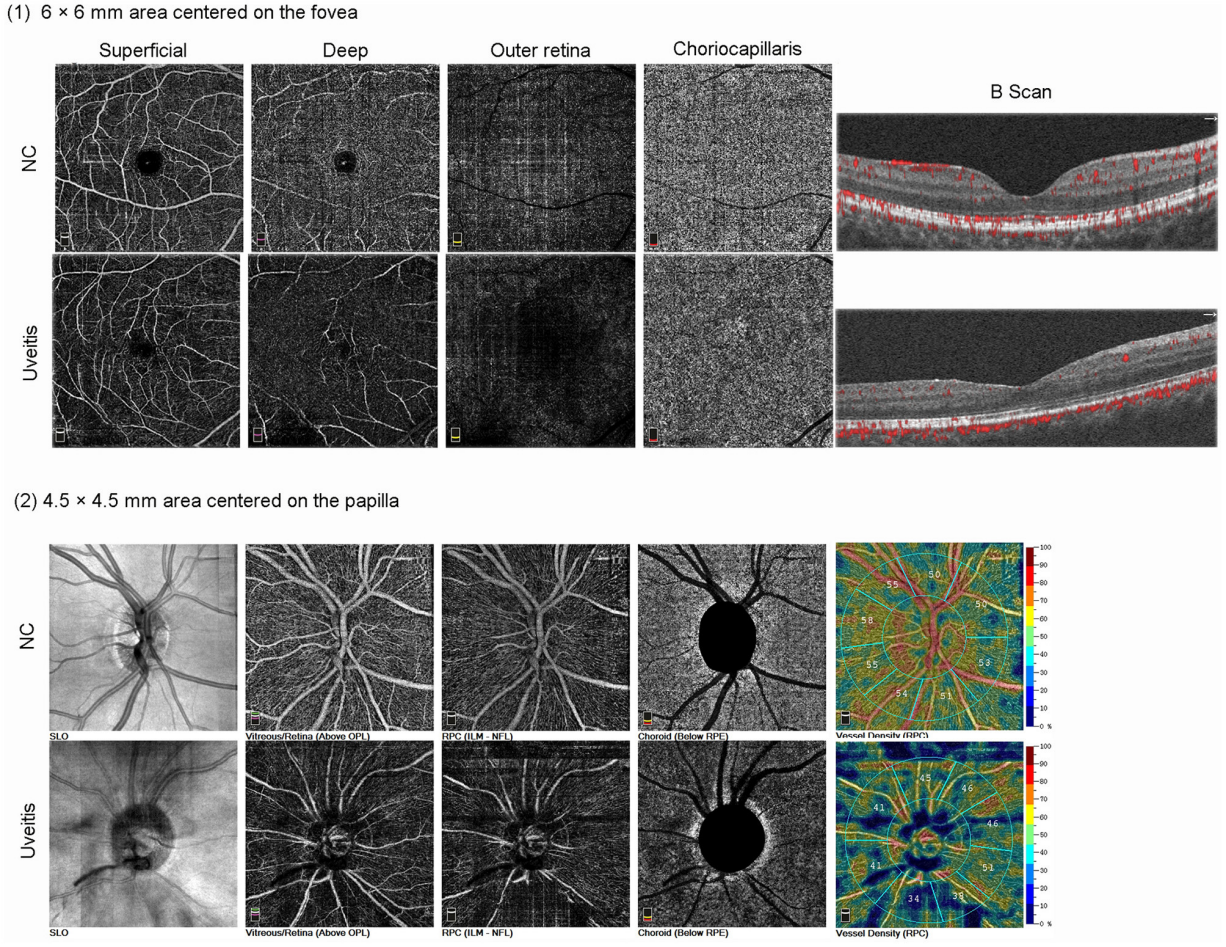
Optical coherence tomography angiography images of the parafoveal capillaries and optic disc in normal control (NC) eye and uveitic eye. Columns represent one eye each from one healthy and one uveitic subject. (1) the 6-mm × 6-mm area centered on the fovea scan. (2) the 4.5-mm × 4.5-mm area centered on the papilla scan.

According to the manufacturer's instructions, central macular thickness (CMT) was defined as the vertical distance from the inner limiting membrane (ILM) to the Bruch's membrane (BRM) at the central fovea of macula. RNFL thickness was defined as the vertical distance from the ILM to the nerve fiber layer (NFL) of the peripapillary region. Measurement of the thickness of the retina is according to the early treatment diabetic retinopathy study (ETDRS) grid. The ETDRS grid comprised of three concentric rings: 1 mm center, 1–3 mm (parafovea), and an outer ring of 3–6-mm diameters (perifovea). The FAZ is the retinal capillary free area located in the central fovea. The FAZ area was measured automatically using AngioVue software using a slab from the ILM offset to the outer plexiform layer (OPL) offset. Foveal density 300 μm (FD300) was defined as the blood flow density in a 300-μm width of a double loop around the FAZ. The radial peripapillary capillary plexus was from ILM to NFL. Optic disk vessel density (VD) defined by the percentage of area occupied by OCTA detected vasculature, including inside the disk area and the peripapillary region. SCP was defined as from ILM to 10 μm above internal plexiform layer (IPL), DCP was defined as from 10 μm above IPL to 10 μm below OPL, outer retina was defined as from 10 μm below OPL to 10 μm above BRM, and choriocapillaris was defined as from 10 μm above BRM to 30 μm below BRM (Figure 1). Parafovea was defined as the area outside of a 1 mm × 1 mm circle centered on the fovea in SCP and DCP. Optic disk vessel density (VD) defined by the percentage of the area occupied by OCTA detected vasculature, including inside the disk area and the peripapillary region (Figure 1).

### Statistical Analysis

IBM SPSS Statistics (Armonk, NY, IBM Corp.) for Windows version 22.0 was used for statistical analysis. GraphPad Prism® (GraphPad Software Inc., La Jolla, CA) version 6.01 was used to plot graphs. Chi square test was used to compare the categorical variables. Shapiro Wilk's W-test was used to test the normality of the numerical variables. For comparison between the two groups, the unpaired *t*-test and the Mann–Whitney U-test were used. One-way ANOVA followed by Dunnett's *post hoc* test was used to compare the different test groups with the control as indicated. Multiple kinds of testing were corrected using the Bonferroni method. The correlation between various parameters and LogMAR BCVA was evaluated using Pearson's correlation analysis. Continuous data are shown as the mean ± standard deviation (SD). Statistical significance was set at *p* < 0.05.

## Results

### Study Population Characteristics

Sixty-five patients with autoimmune posterior uveitis (31 females and 34 males; 65 eyes) with a mean age of 40.86 ± 15.96 years and 65 age-matched NCs (31 females and 34 males; 65 eyes) with a mean age of 45.32 ± 12.11 years were included in the study. There was no statistically significant difference between the patients with uveitis and NCs in terms of age and sex (*p* = 0.118 and *p* = 0.992, respectively). The demographic and clinical characteristics of study groups are summarized in Table 1. In the uveitis group, there were 40 cases of idiopathic panuveitis (*n* = 40; 61.5%), 15 cases of Vogt-Koyanagi-Harada disease (*n* = 15; 23.1%), and 10 cases of Behçet's (*n* = 10; 15.4%).

**Table 1:** Statistical comparison of demographic characteristics of study participants.

	**Normal control (NC)**	**Autoimmune posterior uveitis**	**Acute uveitis**	**Chronic uveitis**	***p*** **-value**
Number of eye/individuals	65/65	65/65	32/32	33/33	
Age (years)	40.86 ± 15.96	45.32 ± 12.11	42.75 ± 19.22	39.03 ± 12.02	0.118
Sex, female/male	31/34	31/34	15/17	16/17	0.992
BCVA(logMAR)	0.24 ± 0.15	0.01 ± 0.17	/	/	<0.001

The mean logMAR BCVA between the patients with uveitis (0.24 ± 0.15) and NCs (0.01 ± 0.17) was different (*p* < 0.001). OCTA images with quality indices of Q7 or above were included. In total, 65 OCTA images of the affected eyes and 65 images of NCs were analyzed. Among them, 32 patients and 32 eyes (32 images), as well as 33 patients and 33 eyes (33 images), were classified into the acute and chronic eye groups.

### Comparison of Fundus Fluorescein Angiography and OCTA Technique in Assessment of Uveitis

**Figure 2:**
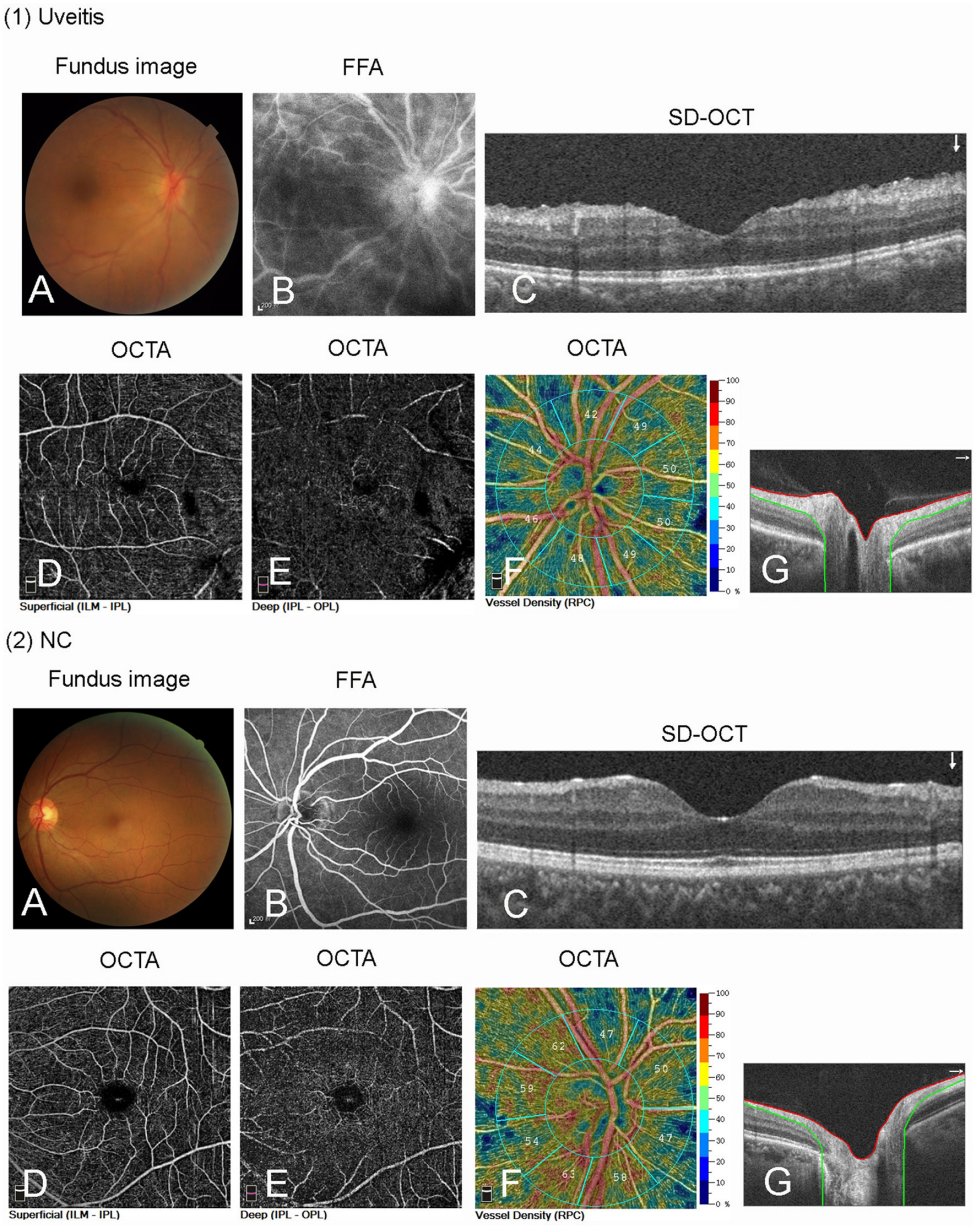
(1) Fundus images of a patient with autoimmune posterior uveitis using different techniques. **(A)** Fundus photography shows optic disk hyperemia and edema, and the absence of foveal reflex. **(B)** Fundus fluorescein angiography (FFA) shows optic disk staining, capillary dilation, and leakage. **(C)** Optical coherent tomography (OCT) shows the thickening of retinal neuroepithelial layer in the macular area. **(D–F)** Optical coherence tomography angiography (OCTA) scan shows decrease of VDs in the superficial capillary plexus (SCP) and deep capillary plexus (DCP) of retina and optic disk. **(G)** The OCTA optic disc scan shows thickening optic disc. (2) Fundus images of a normal control (NC) eye.

Fundus Fluorescein Angiography is a very sensitive imaging method for detecting retinal vascular inflammation, because even slight inflammation of the retinal vascular wall may cause vascular leakage. FFA leakage is a very useful feature for assessing potential uveitis activity. OCTA cannot detect leakage, but it can describe changes in the blood vessel density of the different layers of retina such as superficial or deep capillary plexus, and optic disk. These results indicate that OCTA may be used to quantitatively measure the degree of intraocular inflammation (Figure 2).

### Comparison of OCTA Data in Uveitis Eyes and Normal Control Eyes

The comparisons of RNFL, CMT, retinal thickness, FAZ, FD, and VDs in the optic disk, SCP, DCP, outer retina, and choriocapillaris between uveitis eyes and NCs are summarized in Table 2. As compared to normal eyes, RNFL, CMT, retinal thickness (1–3) μm, retinal thickness (3–6) μm, and retinal thickness (0–6) μm were increased in uveitis eyes (all *p* < 0.05). The FAZ area had no significant change between uveitis and NC (*p* = 0.07). FD-300 Area Density and FD-300 Length Density were decreased in uveitis eyes (all *p* < 0.001). VD in the optic disk was 53.08 ± 5.49 and 56.28 ± 2.51 in uveitis eyes and normal eyes, respectively. SCP was 46.11 ± 4.98 and 49.01 ± 3.83 in the whole image and 46.82 ± 5.29 and 50.79 ± 5.29 in parafovea in uveitis eyes and normal eyes, respectively. DCP was 45.63 ± 5.25 and 48.36 ± 6.53 in the whole image and 51.76 ± 5.71 and 54.19 ± 4.65 in parafovea in uveitis eyes and normal eyes, respectively. Outer retina was 14.72 ± 3.75 and 14.59 ± 3.15 in uveitis eyes and normal eyes, respectively. Choriocapillaris was 23.69 ± 1.59 and 24.22 ± 1.16 in the uveitis eyes and normal eyes, respectively. As compared to normal eyes, VDs in the optic disk, SCP, and DCP both in whole image and parafovea, choriocapillaris were significantly decreased in uveitis eyes (*p* < 0.001, *p* < 0.001, *p* < 0.001, *p* = 0.009, *p* = 0.009, and *p* = 0.031, respectively). Differences in VDs in the outer retina between uveitis eyes and normal eyes were not significant (*p* = 0.826).

**Table 2:** The RNFL, CMT, retinal thickness, FAZ, FD-300, and VDs in the optic disk, SCP, DCP, out retina, and choriocapillaris in uveitis eyes and normal eyes.

	**Uveitis eye**	**Normal eye**	***p*** **-value**
RNFL (μm)	120.80 ± 24.07	101.20 ± 10.01	<0.001
CMT (μm)	264.60 ± 56.16	240.90 ± 20.58	<0.001
Retinal thickness (1–3) μm	338.30 ± 25.32	323.0 ± 10.31	0.001
Retinal thickness (3–6) μm	308.00 ± 27.28	281.70 ± 11.52	<0.001
Retinal thickness (0–6) μm	313.70 ± 28.27	290.10 ± 10.38	<0.001
FAZ (mm^2^)	0.31 ± 0.09	0.33 ± 0.13	0.248
FD-300 area density	47.62 ± 7.50	54.02 ± 5.61	<0.001
FD-300 length density	9.92 ± 2.71	11.55 ± 2.25	<0.001
Optic disk VD	53.08 ± 5.49	56.28 ± 2.51	<0.001
Whole VD SCP (%)	46.11 ± 4.98	49.01 ± 3.83	<0.001
Parafovea VD SCP (%)	46.82 ± 5.29	50.79 ± 5.29	<0.001
Whole VD DCP (%)	45.63 ± 5.25	48.36 ± 6.53	0.009
Parafovea VD DCP (%)	51.76 ± 5.71	54.19 ± 4.65	0.009
VD outer retina (%)	14.72 ± 3.75	14.59 ± 3.15	0.826
VD Choriocapillaris (%)	23.69 ± 1.59	24.22 ± 1.16	0.031

*The data were presented as mean ± SD. SD, standard deviation; CMT, central macular thickness; FAZ, foveal avascular zone; VD, vascular density; FD300, Foveal density 300 μm; SCP, superficial capillary plexus; DCP, deep capillary plexus*.

### Analyses of Correlations Between the BCVA and Quantitative Parameters From OCTA

BCVA was recorded in the logarithm of the minimum angle of resolution (logMAR) units. Correlation analyses between LogMAR BCVA and the quantitative parameters from OCTA are shown in Table 3. In uveitis eyes, LogMAR BCVA was statistically significant and correlated with VDs of whole and parafovea SCP, and whole and parafovea DCP, outer retina. However, the RNFL, CMT, retinal thickness (0–6) μm, FAZ area, FD300, and VDs of optic disk, choriocapillaris were not correlated with visual acuity (all *p* > 0.05). In normal eyes, none of them were correlated with LogMAR BCVA (all *p* > 0.05).

**Table 3:** Correlation between BCVA and quantitative analysis in OCTA.

		**r**	***p*** **-value**
Uveitis eye	RNFL (μm)	0.009	0.944
	CMT (μm)	−0.015	0.903
	Retinal thickness (0–6) μm	0.186	0.139
	FAZ (mm^2^)	0.033	0.797
	FD-300 area density (%)	−0.099	0.431
	FD-300 length density (%)	−0.012	0.921
	Optic disk VD (%)	−0.068	0.585
	Whole VD SCP (%)	−0.375	<0.001
	Parafovea VD SCP (%)	−0.306	<0.001
	Whole VD DCP (%)	−0.437	<0.001
	Parafovea VD DCP (%)	−0.34	0.005
	VD outer retina (%)	0.472	<0.001
	VD choriocapillaris (%)	−0.079	0.527
Normal eye	RNFL (μm)	−0.119	0.341
	CMT (μm)	−0.088	0.482
	Retinal thickness (0–6) μm	−0.079	0.531
	FAZ (mm^2^)	0.025	0.844
	FD-300 area density (%)	−0.001	0.996
	FD-300 length density (%)	−0.041	0.748
	Optic disk VD (%)	−0.045	0.718
	Whole VD SCP (%)	−0.123	0.331
	Parafovea VD SCP (%)	−0.099	0.432
	Whole VD DCP (%)	−0.201	0.109
	Parafovea VD DCP (%)	−0.201	0.095
	VD outer retina (%)	0.014	0.913
	VD Choriocapillaris (%)	0.071	0.572

*BCVA, best-corrected visual acuity; OCTA, optical coherence tomography angiography; CMT, central macular thickness; FAZ, foveal avascular zone; FD300, Foveal density 300 μm; VD, vascular density; SCP, superficial capillary plexus; DCP, deep capillary plexus*.

### Subgroup Analysis of Patients With Uveitis

Uveitis eyes were next divided into an acute group and chronic group. Table 4 shows that, compared with NC eyes, both the acute and the chronic groups had significantly increased RNFL, CMT, retinal thickness (1–3) μm, retinal thickness (3–6) μm, and retinal thickness (0–6) μm. FD-300 Area Density (*p* < 0.001 and *p* < 0.001, respectively) and FD-300 Length Density (*p* < 0.001 and *p* = 0.017, respectively) were decreased in the acute group and the chronic group. Whole SCP VD (*p* < 0.001 and *p* = 0.047, respectively), parafovea SCP VD (both *p* < 0.001), DCP VD (*p* = 0.017 and *p* = 0.042, respectively), and parafovea DCP VD (*p* = 0.030 and *p* = 0.020) were statistically significantly decreased between the acute group vs. the NC group or the chronic group vs. the NC group.

**Table 4:** The comparisons of RNFL, CMT, retinal thickness, FAZ, FD-300, and VDs in the optic disk, SCP, DCP, outer retina, and choriocapillaris among acute and chronic uveitis eyes and normal eyes.

	**Acute uveitis**	**Chronic uveitis**	**Normal**	***p*** **-value**	***p*** **: Acute-Chronic**	***p*** **: Acute-Normal**	***p*** **: Chronic-Normal**
RNFL (μm)	123.70 ± 21.86	117.10 ± 25.69	102.02 ± 10.01	<0.001	0.273	<0.001	<0.001
CMT (μm)	281.90 ± 64.94	254.10 ± 37.08	240.90 ± 20.58	<0.001	0.039	0.026	<0.001
Retinal thickness (1–3) μm	338.40 ± 34.04	338.20 ± 37.14	323.00 ± 10.31	0.004	0.062	0.002	0.001
Retinal thickness (3–6) μm	310.80 ± 32.62	306.50 ± 22.03	281.70 ± 11.52	<0.001	0.537	<0.001	<0.001
Retinal thickness (0–6) μm	322.60 ± 29.61	304.50 ± 23.94	290.10 ± 10.38	<0.001	0.009	<0.001	<0.001
FAZ (mm^2^)	0.27 ± 0.07	0.33 ± 0.09	0.33 ± 0.13	0.060	0.823	0.070	0.150
FD-300 area density	47.25 ± 5.80	47.14 ± 8.62	54.02 ± 5.61	<0.001	0.953	<0.001	<0.001
FD-300 Length Density	9.58 ± 2.44	10.24 ± 2.95	11.55 ± 2.26	<0.001	0.332	<0.001	0.017
Optic disk VD	53.00 ± 5.82	53.16 ± 5.24	56.28 ± 2.52	0.001	0.908	0.001	0.001
Whole VD SCP (%)	44.15 ± 5.45	47.43 ± 3.82	49.01 ± 3.83	<0.001	0.006	<0.001	0.047
Parafovea VD SCP (%)	46.51 ± 6.06	47.13 ± 4.49	50.79 ± 5.29	<0.001	0.006	<0.001	0.001
Whole VD DCP (%)	45.21 ± 4.69	46.04 ± 5.77	48.36 ± 6.53	0.031	0.529	0.017	0.042
Parafovea VD DCP (%)	52.05 ± 4.15	51.42 ± 6.91	54.19 ± 4.65	0.026	0.655	0.030	0.020
VD outer retina (%)	16.10 ± 3.62	13.39 ± 3.41	14.59 ± 3.15	0.005	0.002	0.037	0.086
VD Choriocapillaris (%)	23.31 ± 1.39	24.10 ± 1.72	24.22 ± 1.16	0.009	0.046	<0.001	0.675

*The data were presented as mean ± SD. SD, standard deviation; CMT, central macular thickness; FAZ, foveal avascular zone; VD, vascular density; FD300, Foveal density 300 μm; SCP, superficial capillary plexus; DCP, deep capillary plexus*.

However, only the acute group had decreased VDs of the outer retina (*p* = 0.037) and choriocapillaris (*p* < 0.001) compared with the NC group. In contrast, no significant differences were observed in the FAZ area in the acute group and the chronic group when compared with NC (*p* > 0.05).

As compared to the chronic group, the acute group had a significantly decreased VDs of whole and parafovea SCP (both *p* = 0.006), outer retina (*p* = 0.002), and choriocapillaris (*p* = 0.046).

## Discussion

Uveitis is a general term for inflammation of iris, ciliary body, choroid, and retina tissue. This disease is a common ophthalmic disease that can cause some serious complications and sequelae, and it is one of the main causes of blindness worldwide (17–19). Based on the effect of intraocular inflammation on the pathological changes of blood vessels, fundus angiography tools, including fundus fluorescein angiography and indocyanine green angiography (ICGA), are important in evaluating uveitis. However, angiography is invasive, with certain safety risks, and some patients are allergic to the contrast media. In addition, it cannot be used for quantitative assessment. Seeking for a new, objective, and quantitative method to estimate retinal and choroidal microvascular changes is necessary for medical development.

OCTA is able to evaluate inflammatory eye diseases as vascular changes in the iris, choroid, and retina play an important role in the pathophysiology of ocular inflammation (20). OCTA has been used for the visualization and characterization of perifoveal microvascular changes in several kinds of uveitis. Recently, it has also provided quantitative analysis for retinal vascular flow abnormalities, which help demonstrate microvascular changes accompanied with uveitis (1, 21, 22). OCTA analysis has the advantage of: (1) detecting early microvascular changes, which might be beneficial for early diagnosis; (2) quantitatively evaluate the disease status before and after treatment. Similar to Spectral domain optical coherence tomography, OCTA can also measure retinal thickness in different layers, such as RNFL, CMT, and retinal thickness (0–6) μm. Although several previous studies using OCTA have identified vascular flow abnormalities in patients with uveitis, the vascular beds below the RPE complex have not been thoroughly investigated, and quantitative analysis of optic disk, outer retina, and choriocapillaris vascular abnormalities in posterior uveitis is limited. In addition, fewer studies explore the correlation between BCVA and vascular flow in patients with uveitis. In the present study, we aimed to measure vascular flow in different layers, including optic disk, whole SCP, parafovea SCP, whole DCP, parafovea DCP, outer retina, and choriocapillaris.

Our research revealed that patients with posterior uveitis had a significant reduction of VDs in the whole and parafovea SCP and the whole and parafovea DCP compared to healthy controls (*p* < 0.05). Consistent with previous findings, the patients with Behçet's disease and VKH, both VDs, were reported to present decreased SCP and DCP compared to healthy controls (1, 15). A reduction of VDs in the SCP and DCP detected by OCTA in birdshot chorioretinopathy has also been reported (23). It was also found in children with anterior uveitis that the VDs in the SCP and DCP were significantly reduced when compared with NCs, suggesting anterior segment inflammation in pediatric uveitis is associated with reduced retinal vascular density (22). In this study, we not only analyzed the blood flow density of SCP and DCP in a more detailed way but also added some new parameters, such as VDs of the optic disk, outer retina, and choriocapillaris, as well as FD300. FD300 is the foveal blood flow density within a width of 300μm around the FAZ area. We found that VDs in the optic disk, FD300, and choriocapillaris had significantly decreased when compared with NC, while VD in the outer retina had no significant change. We found whole SCP VD, parafovea SCP VD, DCP VD, and parafovea DCP VD were statistically significantly decreased between the acute group vs. the NC group or the chronic group vs. the NC group. In addition, VDs of the optic disk, FD300, were significantly decreased both in the acute group and the chronic group when compared with the NC group, respectively. However, only the acute group had decreased VDs of the outer retina (*p* = 0.037) and choriocapillaris (*p* < 0.001) compared with the NC group. It is possible because the patients in the acute phase had more severe inflammation than in the chronic phase (1). All this evidence indicates that blood flow impairment might be a result of inflammation.

We should carefully interpret the above findings. Intraocular inflammation, regardless of localization, can induce macular edema. This is thought to be the result of a breakdown of the inner and outer blood–retinal barrier and due to inflammatory structural changes, leading to disturbed ocular blood flow (24). Since retinal edema could produce masking artifacts that lead to artificially reduced flow signals from the retina, it is not possible to attribute causality of these vascular changes to either macular edema or uveitis alone solely based on this cross-sectional study, since a longitudinal analysis following patients before, during, and after the development of retinal edema was not performed using OCTA in our study. Future studies should take macular edema into account for the analysis.

The FAZ is largely responsible for visual acuity and central vision. Its size reflects the health of retinal microcirculation (25). Therefore, the measurement of FAZ plays an important role in the diagnosis and management of retinal vascular diseases (6, 26). Interestingly, in our study, the patients with uveitis showed no significant difference from NC in the mean area of FAZ (*p* = 0.248). Waizel et al. reported that eyes suffering from non-infectious posterior uveitis presented significantly larger deep FAZ when compared to healthy controls (26). On the contrary, in multiple evanescent white dot syndrome (MEWDS) eyes and birdshot chorioretinopathy, significantly enlarged FAZ was found (7, 12). It has also been reported that the FAZ area was not different between patients with Behçet's and the control group (15). Therefore, the clinical application of FAZ to indicate pathological changes is controversial. In addition, the area of FAZ in healthy people is highly variable due to individual differences, partly because of the impact of the ocular axis and other factors (27–29). Instead, we found that FD300 decreased significantly. One explanation for this finding is that FD300 is more accurate in reflecting the ischemic degree of fovea, that is, a more sensitive marker than FAZ. FD300 may be a more valuable marker than the FAZ area for detecting pathology in patients with autoimmune posterior uveitis.

Uveitis sometimes causes irreversible visions loss; we analyzed the relationship between LogMAR BCVA and all the quantitative results from OCTA. We found that logMAR BCVA was negatively correlated with VDs in the whole and parafovea SCP, whole and parafovea DCP, and outer retina, while there were no correlations between logMAR BCVA and RNFL, CMT, retinal thickness, FAZ, FD30, and VD in the optic disk, choriocapillaris in patients with uveitis. In addition, whole DCP was the best predictive factor for BCVA in the patients with uveitis. One possible explanation is that DCP is closer to the choroid. The location of the watershed makes it more susceptible to inflammation (7). Similar findings were also found in VKH, in which DCP VD was significantly correlated with visual acuity (1). OCTA is a promising indicator for uveitis prognosis and treatment guidance.

On the other hand, structural measurements of all retinal layers could be provided by OCTA. In the present study, RNFL, CMT, and retinal thickness were found to be significantly thicker in patients with uveitis than in healthy controls. A thicker RNFL in patients with uveitis had been reported. Similar to our study, Yilmaz et al. found RNFL was significantly thicker in patients with uveitis; patients with acute uveitis had a thicker RNFL than the NCs and the patients with quiescent uveitis (30). The difference in CMT and retinal thickness was found to be statistically significant in patients with uveitis and healthy controls. Ataş et al. found out that macular thickness was thinner in patients with Behçet's disease than in the healthy control group (31). However, previous studies were assessed by optical coherence tomography but not by OCTA.

Our study had some limitations: for example, the overall sample size was modest. Even so, statistically significant differences were found between uveitis and NC. In addition, there is no more detailed subgroup analysis according to the etiology of uveitis. Considering all of the above, we believe that further studies should be carried out to evaluate the microvasculature by OCTA in a variety of autoimmune posterior uveitis with different etiology, such as Behçet's disease, and VKH.

In conclusion, this was a clinical OCTA-based investigation of the microvascular changes associated with autoimmune posterior uveitis and posterior uveitis. Our results demonstrated a novel finding that VDs of optic disk, retinal, and choroidal decreased, and that perifoveal microvascular network changed in patients with uveitis. Quantitative measurement by OCTA demonstrates good ability for detecting pathological changes and is a potential tool to assist the diagnosis and inflammation evaluation in patients with autoimmune posterior uveitis.

## Data Availability Statement

The raw data supporting the conclusions of this article will be made available by the authors, without undue reservation.

## Ethics Statement

The study was approved by the Ethics Committee of the Second Affiliated Hospital, School of Medicine, Zhejiang University. The patients/participants provided their written informed consent to participate in this study. Written informed consent was obtained from the individual(s) for the publication of any potentially identifiable images or data included in this article.

## Author Contributions

JS: conception, design, image interpretation, and manuscript preparation. JK and SC: manuscript preparation and data collection. XL, YT, HW, LW, and MW: patient treatment and data collection. ZS and LF: conception and design. All authors have read and approved the final manuscript.

## Funding

This study was supported in part by the National Natural Science Foundation of China (Nos. 81870648, 82070949, and 82000886) and Natural Science Foundation of Zhejiang Province (No. LQ20H120011).

## Conflict of Interest

The authors declare that the research was conducted in the absence of any commercial or financial relationships that could be construed as a potential conflict of interest.

## Publisher's Note

All claims expressed in this article are solely those of the authors and do not necessarily represent those of their affiliated organizations, or those of the publisher, the editors and the reviewers. Any product that may be evaluated in this article, or claim that may be made by its manufacturer, is not guaranteed or endorsed by the publisher.
